# Hearing impairment and associated morphological changes in pituitary adenylate cyclase activating polypeptide (PACAP)-deficient mice

**DOI:** 10.1038/s41598-019-50775-z

**Published:** 2019-10-10

**Authors:** Daniel Balazs Fulop, Viktoria Humli, Judit Szepesy, Virag Ott, Dora Reglodi, Balazs Gaszner, Adrienn Nemeth, Agnes Szirmai, Laszlo Tamas, Hitoshi Hashimoto, Tibor Zelles, Andrea Tamas

**Affiliations:** 1 Department of Anatomy, MTA-PTE PACAP Research Team, Centre for Neuroscience, University of Pecs Medical School, Pecs, Hungary; 2 Department of Pharmacology and Pharmacotherapy, Semmelweis University, Budapest, Hungary; 3 Department of Otorhinolaryngology, University of Pecs Medical School, Pecs, Hungary; 4 Department of Otorhinolaryngology, Head and Neck Surgery, Semmelweis University, Budapest, Hungary; 5 Laboratory of Molecular Neuropharmacology, Graduate School of Pharmaceutical Sciences, Osaka University, Suita, Osaka Japan; 6 Molecular Research Center for Children’s Mental Development, United Graduate School of Child Development, Osaka University, Kanazawa University, Hamamatsu University School of Medicine, Chiba University and University of Fukui, Suita, Osaka Japan; 7 Division of Bioscience, Institute for Datability Science, Osaka University, Suita, Osaka Japan; 8 Department of Pharmacology, Institute of Experimental Medicine, Hungarian Academy of Sciences, Budapest, Hungary

**Keywords:** Cochlea, Transduction, Molecular neuroscience, Mechanisms of disease, Peptides

## Abstract

Pituitary adenylate cyclase activating polypeptide (PACAP) is a regulatory and cytoprotective neuropeptide, its deficiency implies accelerated aging in mice. It is present in the auditory system having antiapoptotic effects. Expression of Ca^2+^-binding proteins and its PAC1 receptor differs in the inner ear of PACAP-deficient (KO) and wild-type (WT) mice. Our aim was to elucidate the functional role of PACAP in the auditory system. Auditory brainstem response (ABR) tests found higher hearing thresholds in KO mice at click and low frequency burst stimuli. Hearing impairment at higher frequencies showed as reduced ABR wave amplitudes and latencies in KO animals. Increase in neuronal activity, demonstrated by c-Fos immunolabeling, was lower in KO mice after noise exposure in the ventral and dorsal cochlear nuclei. Noise induced neuronal activation was similar in further relay nuclei of the auditory pathway of WT and KO mice. Based on the similar inflammatory and angiogenic protein profile data from cochlear duct lysates, neither inflammation nor disturbed angiogenesis, as potential pathological components in sensorineural hearing losses, seem to be involved in the pathomechanism of the presented functional and morphological changes in PACAP KO mice. The hearing impairment is probably concomitant with the markedly accelerated aging processes in these animals.

## Introduction

Pituitary adenylate cyclase activating polypeptide (PACAP) is a neuropeptide, member of the VIP/glucagon/secretin peptide family showing 68% homology with VIP (vasoactive intestinal peptide). PACAP occurs in two biological active forms, PACAP38 containing 38 amino acids, and PACAP27 consisting of the N-terminal 27 amino acid residue of PACAP38^1^. PACAP has its specific PAC1 receptor, and it also exerts its effects through VPAC1 and VPAC2 receptors which bind PACAP and VIP with equal affinity^2^. PACAP is abundant in the central and peripheral nervous system having neurotrophic and neuroprotective effects^3,4^. It is protective in different neurodegenerative and cerebral ischemia models^5–7^. PACAP is also present in several organs exerting antiapoptotic, cytoprotective and regulatory functions^2^. These regulatory functions in basic physiological processes are also supported by its evolutionary well-conserved sequence among species^2,8^. PACAP and its receptors are expressed in neuroepithelial cells at an early stage of ontogenesis^9^. It is also known that PACAP affects the development of several other cell types besides neurons, *e.g*. retinoblasts and cells of the olfactory system, indicating that PACAP has important functions in different sensory organs^10,11^. It has well-known retinoprotective effects in different lesions of the retina, which were described morphologically, functionally and at the molecular level^12–14^. PACAP exerts protective functions in the olfactory system^15^. PACAP has also been described in several parts of the auditory system^16^. Briefly, PACAP and PAC1 receptor are present in hair cells, supporting cells, spiral ganglion neurons, afferent and efferent nerve fibres, in the stria vascularis of the cochlea^17–20^ and in the nuclei of the auditory pathway^21,22^. Our previous *in vitro* experiments showed that PACAP protects the inner ear hair cells in case of oxidative stress. In a chicken inner ear cell culture, we applied H_2_O_2_ to induce reactive oxygen species production and we found that the number of living cells was higher and the apoptosis rate lower in case of PACAP co-treatment compared to the control H_2_O_2_-treated group^23^. We also performed *in vivo* studies on PACAP-deficient mice (KO)^24^. Based on developmental functions of PACAP, there are disturbances during the ontogenesis of PACAP-deficient mice compared to wild type (WT) mice and plasticity after injury is also disturbed. PACAP KO mice have no macroscopic differences compared to WT mice, however, they show biochemical changes^25^, behavioural alterations^26,27^, memory disorders^28^ and reduced fertility^29,30^. There are differences in several organ systems, as abnormal axonal arborization in the brain^31^, altered bone and cartilage formation^32^ and tooth development^33,34^. They show higher vulnerability after injuries in the brain, internal organs and the retina^35–37^. Intraperitoneal nitroglycerol treatment results in reduced meningeal blood flow elevation in PACAP KO animals confirming that PACAP is an important mediator of migraine^38^. The lack of endogenous PACAP also leads to accelerated aging in several organ systems^39,40^. For example elevated corneal angiogenesis, accelerated aging of the retina, more severe systemic amyloidosis and impaired antioxidant capacity were found in PACAP KO animals^39,41,42^. We hypothesize that proteins and cytokines playing important roles in angiogenesis [acidic fibroblast growth factor (FGF), C-X-C motif chemokine 12 (CXCL12), endostatin, Serpin F1], inflammatory processes [B lymphocyte chemoattractant (BLC), platelet factor 4 (PF4), CXCL12] and in coagulation [PF4, tissue factor (TF)] could also play an important role in the inner ear functions. Our previous experiments in PACAP-deficient mice showed that the expression of PAC1 receptor in the hair cells and outer phalangeal cells is decreased compared to WT mice^17^. There was an elevation in Ca^2+^-binding protein levels in inner ear hair cells of PACAP KO animals under normal circumstances compared to WT animals that could not be increased by ototoxic kanamycin treatment. These alterations indicate pathological deterioration in PACAP KO animals^17,43^. In our current study, we elucidated the functional outcome of PACAP deficiency on hearing and attempted to clarify some of the underlying molecular mechanisms. We measured in WT and PACAP-deficient mice the hearing thresholds, amplitudes and latencies of auditory brainstem responses (ABRs); quantified the neuronal activation in the nuclei of the auditory pathway after acoustic stimulation; and performed proteome profile analysis of cochlear duct lysates to explore the molecular mechanisms of the impairing effect of endogenous PACAP deficiency on hearing.

## Results

### *In vivo* ABR recordings – Hearing thresholds

**Figure 1:**
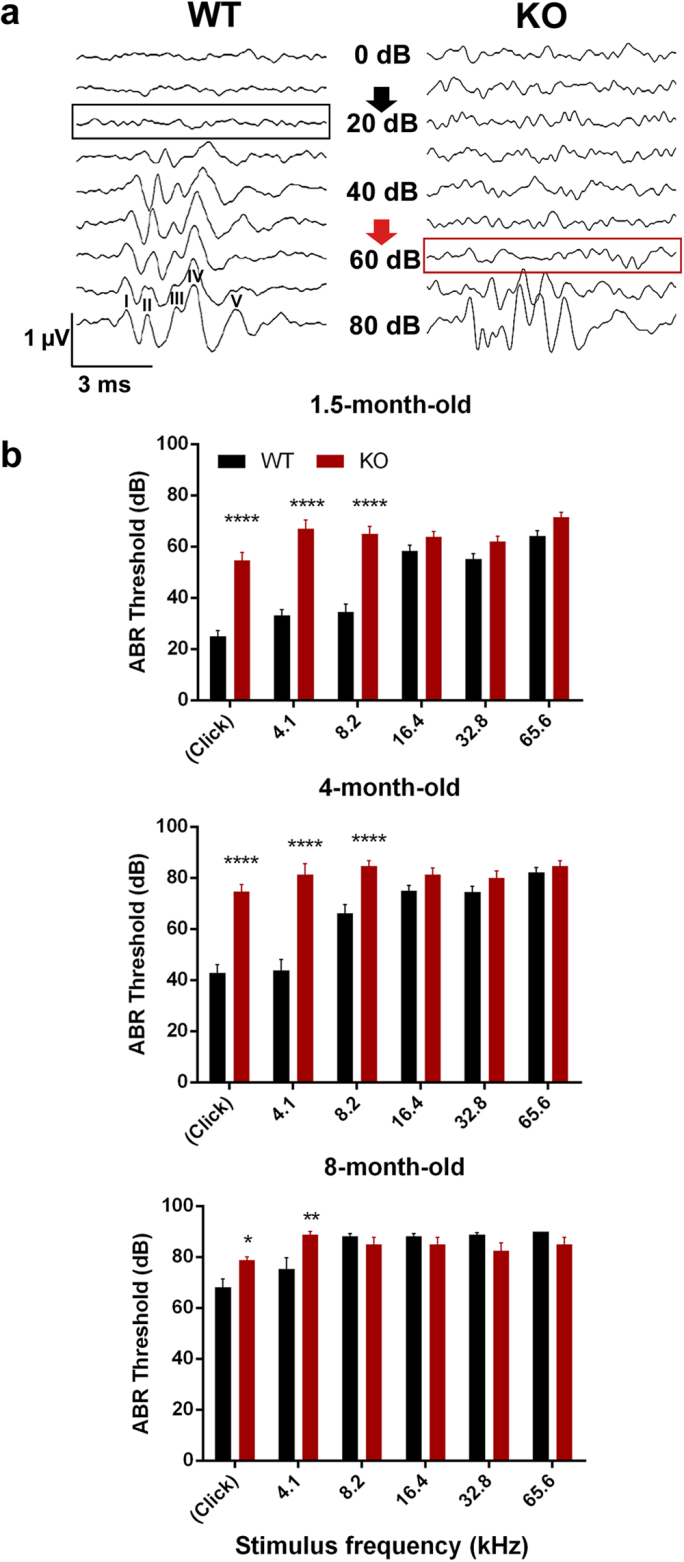
Hearing threshold measurements with auditory brainstem response (ABR) tests in wild-type (WT) and PACAP-deficient (KO) mice. (**a**) Individual curves of evoked potentials (click stimuli) in 1.5-month-old mice. Hearing threshold at 20 dB sound pressure in WT (black arrow) and at 60 dB sound pressure in KO mice (red arrow). ABR peaks I-V labelled at 80 dB WT curve: peak I arising from the cochlea/spiral ganglion neurons, peaks II-V arising from upper nuclei of the auditory pathway. (**b**) Hearing thresholds in WT and KO mice with click stimuli and at different frequencies at the age of 1.5, 4 and 8 months. Asterisks indicate significant differences between KO and WT mice. Mean ± SEM, 2-way-ANOVA, Bonferroni post-hoc test. ****p < 0.0001, **p < 0.01, *p < 0.05 vs. WT values at the corresponding stimulus frequency.

PACAP KO mice had significantly higher hearing thresholds at click and at low frequency burst stimuli – 4.1 kHz and 8.2 kHz – at the age of 1.5 months (Fig. 1, Table 1). This represents a massive hearing deficit in PACAP KO mice compared to the WT animals. We repeated the ABR measurements at the age of 4 and 8 months (Fig. 1/b, Table 1). The auditory thresholds were further elevated at lower frequencies in older PACAP KO mice, however, the WT mice also reached a plateau with aging. Therefore, the difference between the two groups was less pronounced (Fig. 1/b, Table 1). At higher frequencies, we did not find differences in the hearing thresholds between the two groups at either age.

**Table 1:** Results of hearing threshold measurements with auditory brainstem response (ABR) tests in wild-type (WT) and PACAP-deficient (KO) mice with different stimuli at 1.5, 4 and 8 months of age. ns = non-significant.

Age	Stimulus	WT (Mean ± SEM)	KO (Mean ± SEM)	p value
1.5 months	click	25 ± 2.33 dB	54.62 ± 3.15 dB	p < 0.0001
1.5 months	4.1 kHz	33.1 ± 2.33 dB	66.92 ± 3.5 dB	p < 0.0001
1.5 months	8.2 kHz	34.48 ± 3.04 dB	65 ± 2.9 dB	p < 0.0001
4 months	click	42.78 ± 3.31 dB	74.67 ± 2.74 dB	p < 0.0001
4 months	4.1 kHz	43.89 ± 4.13 dB	81.33 ± 4.24 dB	p < 0.0001
4 months	8.2 kHz	66.11 ± 3.44 dB	84.67 ± 2.15 dB	p < 0.0001
8 months	click	68.24 ± 3.12 dB	78.75 ± 1.25 dB	p = 0.0364
8 months	4.1 kHz	75.29 ± 4.47 dB	88.75 ± 1.25 dB	p = 0.003
8 months	8.2 kHz	88.24 ± 0.95 dB	85 ± 2.67 dB	ns

### *In vivo* ABR recordings – Analysis of peak amplitudes and latencies

**Figure 2:**
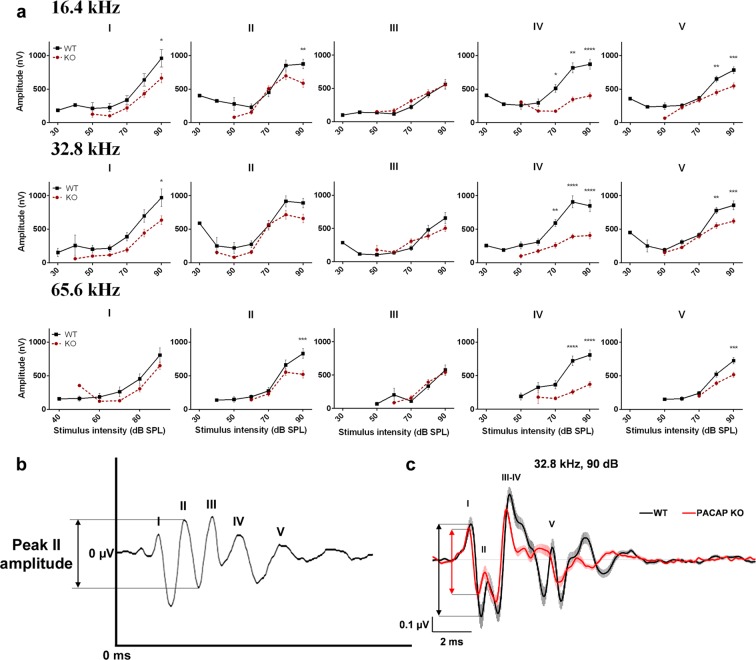
Genotype-dependent differences in the auditory brainstem response (ABR) amplitude-intensity profiles of PACAP-deficient (KO) and wild-type (WT) mice at 16.4, 32.8 and 65.6 kHz. (**a**) Asterisks indicate significant differences between KO and WT mice. Mean ± SEM; 2-way-ANOVA, Bonferroni post-hoc test. ****p < 0.0001, ***p < 0.001, **p < 0.01, *p < 0.05 vs. WT values at the corresponding stimulus frequency and intensity. (**b**) Example for absolute amplitude measurement at peak II. (**c**) Average traces (±SEM) at 32.8 kHz, 90 dB demonstrate the waveform differences between WT and KO mice. Two-headed arrows (↕) demonstrate the difference between peak I amplitude values (black: WT, red: KO).

**Figure 3:**
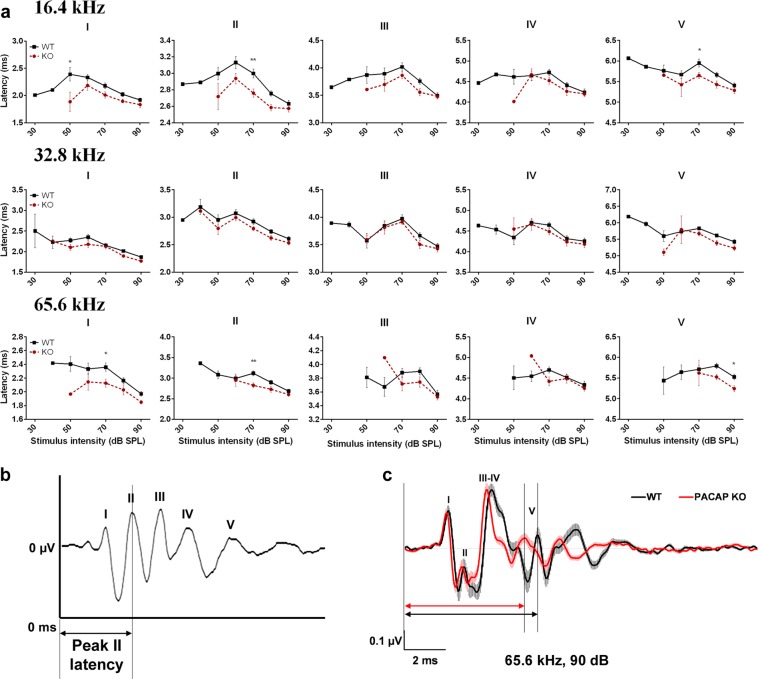
Genotype-dependent differences in the auditory brainstem response (ABR) latency-intensity profiles of PACAP-deficient (KO) and wild-type (WT) mice at 16.4, 32.8 and 65.6 kHz. (**a**) Asterisks indicate significant differences between KO and WT mice. Mean ± SEM; 2-way-ANOVA, Bonferroni post-hoc test. **p < 0.01, *p < 0.05 vs. WT values at the corresponding stimulus frequency and intensity. (**b**) Example for absolute latency measurement at peak II. (**c**) Average traces (±SEM) at 65.6 kHz, 90 dB demonstrate the waveform and latency differences between WT and KO mice. Two-headed arrows (↔) demonstrate the difference between peak V latency values (black: WT, red: KO).

**Figure 4:**
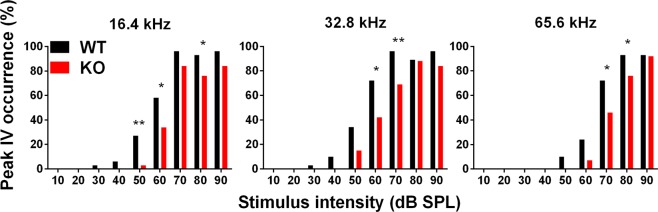
Proportions (%) of separate auditory brainstem response (ABR) peak IV at increasing intensity levels at 16.4, 32.8 and 65.6 kHz tone burst stimuli. In wild-type (WT) mice, peak IV is more identifiable, than in PACAP-deficient (KO) mice. ‘N-1’ Chi-squared test, **p < 0.01, *p < 0.05 vs. WT values at the corresponding stimulus frequency and intensity.

We analysed the ABR wave amplitudes and latencies in 1.5-month-old mice at all measured stimulus intensity levels to explore further differences between the PACAP KO and WT mice at higher frequencies (16.4, 32.8 and 65.6 kHz) where there were no differences in the hearing thresholds (Figs 2, 3). All WT and PACAP KO mice were analysed, but not all 5 peaks appeared at each frequency at each sound pressure level [e.g. peak I at 16.4 kHz presented only in one WT mice at 30 dB sound pressure level (SPL), while in 29 WT mice at 90 dB SPL]. There was a significant decrease in the ABR peak amplitudes of PACAP KO mice on peaks I-II and IV-V at high stimulus intensity (Fig. 2, for P values please see Supplementary Table S1). The latencies were also tendentially shorter at high frequencies in all peaks and the differences were significant in several cases (Fig. 3, for P values please see Supplementary Table S1). Differences are easily recognizable visually on the superimposed average curves of ABR waves of the PACAP KO and WT mice at 32.8 and 65.6 kHz (Figs 2/c and 3/c). We often observed the fusion of peaks III and IV in both WT and PACAP KO animals. However, this merge occurred more frequently in PACAP KO mice, as it is shown by the lower incidence of peak IV in these mice at 16.4, 32.8 and 65.6 kHz at higher stimulus intensities (Fig. 4, Supplementary Table S1).

### Auditory pathway activation and cell count measurement – c-Fos immunolabeling, Nissl staining

**Figure 5:**
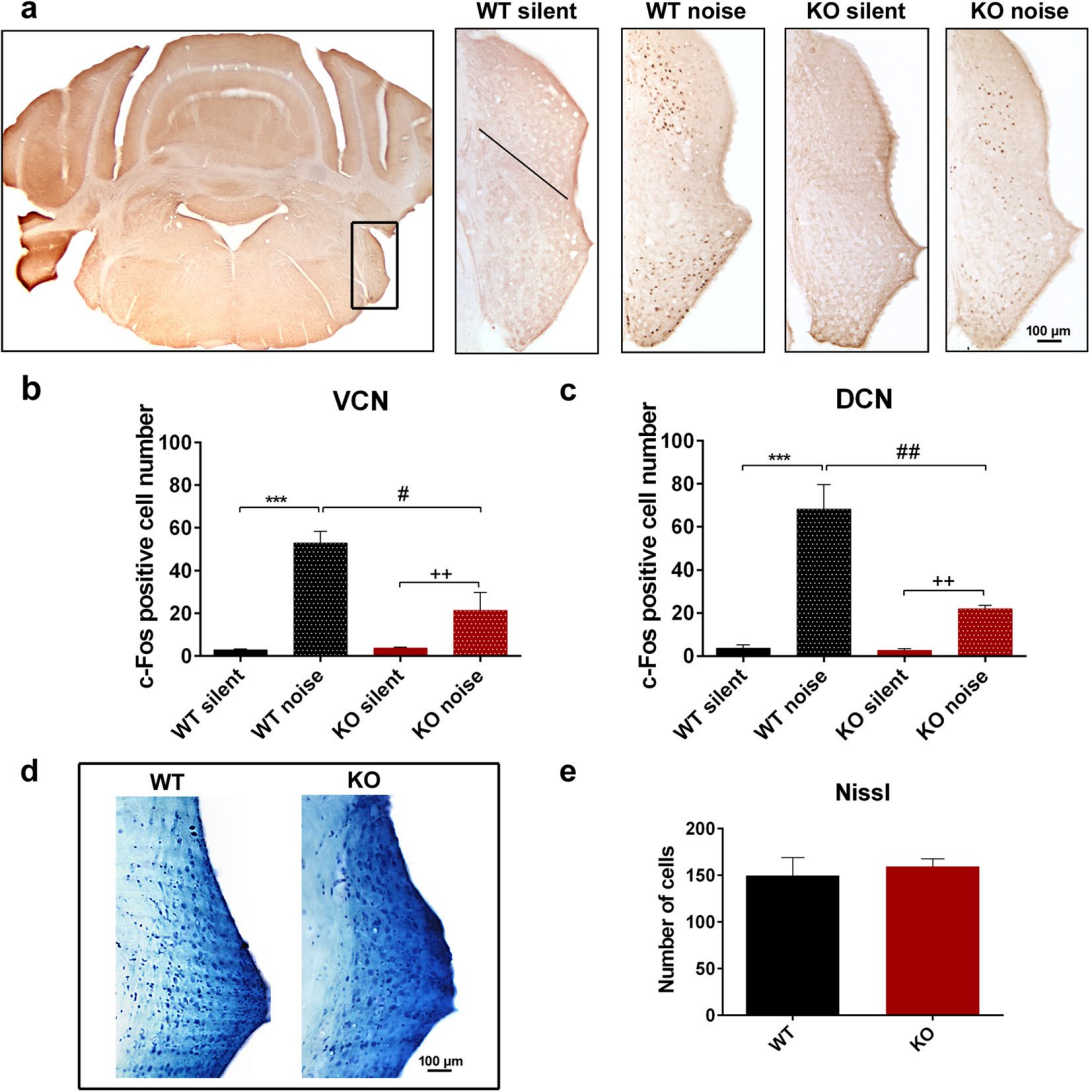
Expression of c-Fos in the dorsal and ventral cochlear nuclei (DCN, VCN) of 1.5-month-old wild-type (WT) and PACAP-deficient (KO) animals after noise exposure (noise) or without noise exposure (silent). (**a**) Representative images with lower and higher magnification of the VCN and DCN (rectangle in the low magnification picture) in frontal sections of the brainstem. Black line in ‘WT silent’ image represents the border between VCN (below) and DCN (above). (**b**,**c**) Number of c-Fos immunopositive cells in VCN and DCN, respectively. Noise application caused a significant elevation of activated neurons in WT and KO mice, but fewer activated neurons were in PACAP KO animals compared to WT mice. Mean ± SEM; 2-way-ANOVA, Bonferroni post-hoc test, ***p < 0.001 vs. WT silent; ^++^p < 0.01 vs. KO silent; ^##^p < 0.01, ^#^p < 0.05 vs. WT noise. (**d**) Representative images of Nissl-staining of VCN and DCN of 4-month-old WT and KO mice. (**e**) Sum of the number of neurons in VCN and DCN in WT and KO mice. There was no significant difference between WT and PACAP KO animals. Mean ± SEM, Student’s t-test.

**Figure 6:**
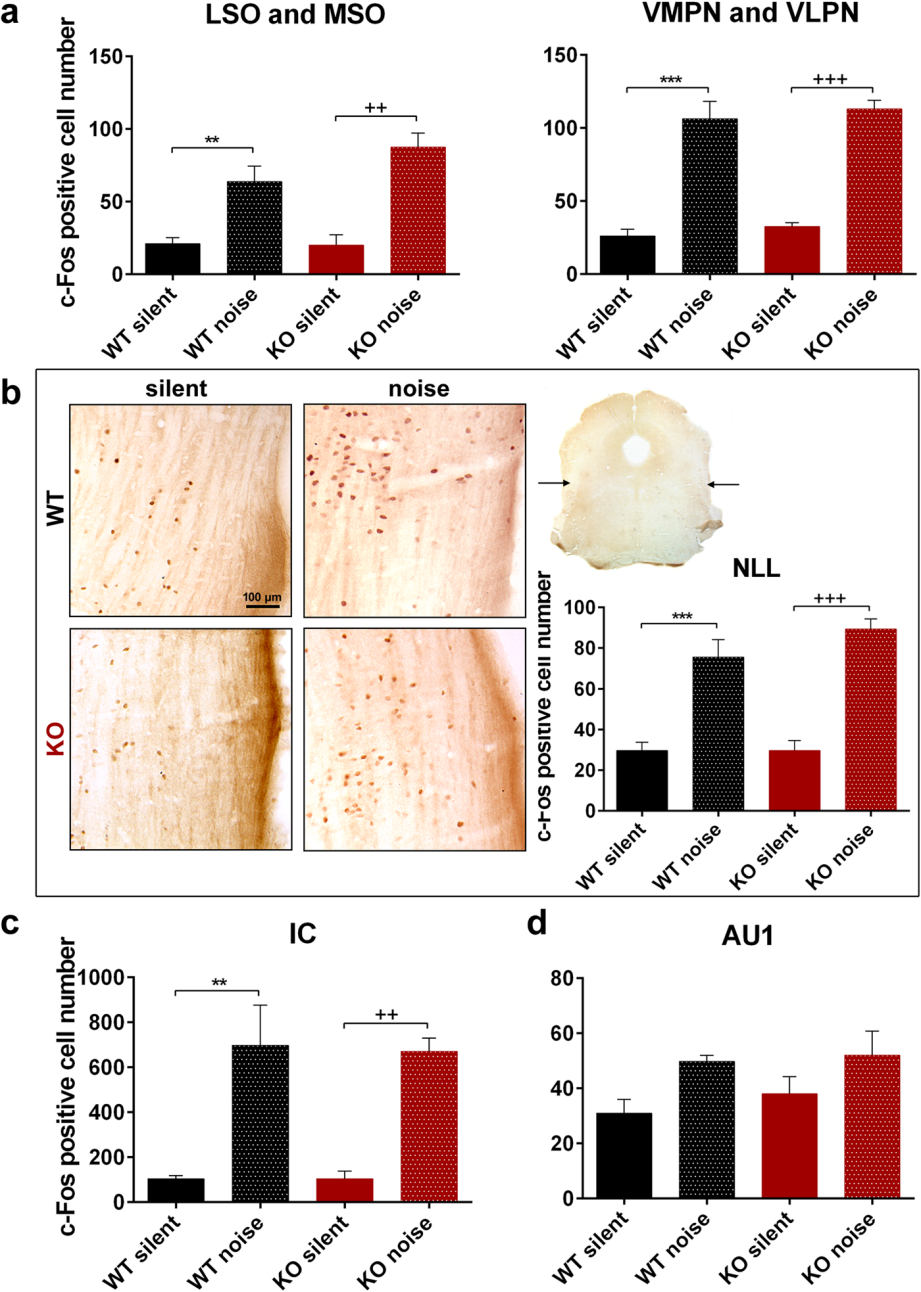
Expression of c-Fos in the central relay nuclei of the auditory pathway of 1.5-month-old wild-type (WT) and PACAP-deficient (KO) mice after noise exposure (noise) or without noise exposure (silent). (**a**) Superior olivary complex: lateral and medial superior olive (LSO, MSO), and ventromedial and ventrolateral periolivary nuclei (VMPN, VLPN). (**b**) Nuclei of the lateral lemniscus (NLL): representative images with lower and higher magnification in frontal sections of the brainstem and numerical analysis. Arrows on the low magnification inset show the location of the dorsal part of the NLL. (**c**) Inferior colliculus (IC). (**d**) Primary auditory cortex (AU1). Noise application caused a significant elevation in the number of activated neurons both in WT and KO mice (non-significant in the AU1), but there was no difference between the two genotypes. (a–d) Mean ± SEM; 2-way-ANOVA, Bonferroni post-hoc test. ***p < 0.001, **p < 0.01 vs. WT silent; ^+++^p < 0.001, ^++^p < 0.01 vs. KO silent.

We examined the neuronal activation with c-Fos immunoreactivity in the auditory pathway (Figs 5, 6, Table 2). In the ventral cochlear nucleus (VCN), there was almost no cell activation in mice, which were held in a silent environment for 24 hours before termination (labelled as ‘silent’). There was a significant elevation of activated neurons in both WT and PACAP KO animals after exposure to half an hour filtered white noise. The elevation was significantly smaller in KO animals compared to WT ones (Fig. 5/a,b, Table 2). We found similar results in the dorsal cochlear nucleus (DCN) with almost no cell activation in the silent environment and significant elevation in the number of activated neurons after noise application both in WT and PACAP KO animals. The elevation was significantly smaller in KO animals compared to WT animals (Fig. 5/a,c, Table 2). We applied Nissl staining to the slides of VCN and DCN of WT and PACAP KO animals to decide whether a decreased number of cells had caused the reduced c-Fos immunopositivity in PACAP KO animals. Despite the different cell activation with c-Fos immunostaining there was no difference in cell number in the VCN and DCN between the WT and PACAP KO animals (WT = 149.88 ± 18.99 and KO = 159.50 ± 8.00) (Fig. 5/d,e).

**Table 2:** Expression of c-Fos in the nuclei of the auditory pathway of 1.5-month-old wild-type (WT) and PACAP-deficient (KO) animals after noise exposure (noise) or without noise exposure (silent).

Nucleus	WT silent	WT noise	KO silent	KO noise	p value		
					p1	p2	p3
VCN	2.96 ± 0.32	52.95 ± 5.46	3.73 ± 0.36	21.47 ± 8.38	p < 0.0001	p = 0.0012	p = 0.0214
DCN	3.68 ± 1.64	68.23 ± 11.43	2.83 ± 0.72	22 ± 1.73	p < 0.0001	p = 0.0042	p = 0.0012
MSO-LSO	21.24 ± 3.99	63.97 ± 10.54	20.23 ± 7.11	87.6 ± 9.6	p = 0.0096	p = 0.0033	ns
VMPN-VLPN	26.24 ± 4.42	106.38 ± 11.8	32.50 ± 2.84	113.10 ± 5.90	p < 0.0001	p = 0.0007	ns
NLL	29.73 ± 4.16	75.61 ± 8.61	29.73 ± 4.95	89.35 ± 4.90	p < 0.0001	p < 0.0001	ns
IC	104.59 ± 13.8	697.6 ± 178.4	105 ± 33.66	671.33 ± 59.2	p = 0.0019	p = 0.0038	ns
AU1	31.06 ± 4.90	49.85 ± 2.15	38.00 ± 6.21	52.09 ± 8.78	ns	ns	ns

Mean ± SEM. VCN, DCN: ventral and dorsal cochlear nuclei, MSO: medial superior olive, LSO: lateral superior olive, VMPN: ventromedial periolivary nucleus, VLPN: ventrolateral periolivary nucleus, NLL: nuclei of the lateral lemniscus, IC: inferior colliculus, AU1: primary auditory cortex; p1: WT noise vs. WT silent, p2: KO noise vs. KO silent, p3: KO noise vs. WT noise; ns = non-significant.

The differences between the WT and KO animals disappeared in the central parts of the auditory pathway (Fig. 6). There was a small neuronal activation in the silent group regarding the medial superior olive (MSO) and lateral superior olive (LSO) parts of the superior olivary complex (SOC) in both WT and PACAP KO animals. We found significant elevation of c-Fos immunolabeling in both WT and PACAP KO animals after noise application but there was no significant difference between the WT and PACAP KO groups (Fig. 6/a, Table 2). We also measured the number of activated cells in the region of ventromedial and ventrolateral periolivary nuclei (VMPN and VLPN, respectively) with similar results. Noise application resulted in an elevation of activated neurons in both WT and PACAP KO animals but there was no difference between the WT and PACAP KO groups (Fig. 6/a, Table 2). The nuclei of the lateral lemniscus (NLL) yielded similar results: we measured neuronal activation in the silent animal groups that was significantly elevated after noise application but there was no difference between the WT and PACAP KO groups (Fig. 6/b, Table 2). We also found in the inferior colliculus (IC) a baseline cell activation in silent animal groups which elevated significantly after noise application again with no difference between the WT and PACAP KO groups (Fig. 6/c, Table 2). In the primary auditory cortex, the neuronal activation in the silent animal groups showed a tendency to be elevated after noise application but this elevation was not significant. There was no significant difference between the WT and PACAP KO animals (Fig. 6/d, Table 2). Without significant differences in the number of activated neurons, we did not perform Nissl staining in these nuclei.

### Cochlear duct proteome profile analysis

**Figure 7:**
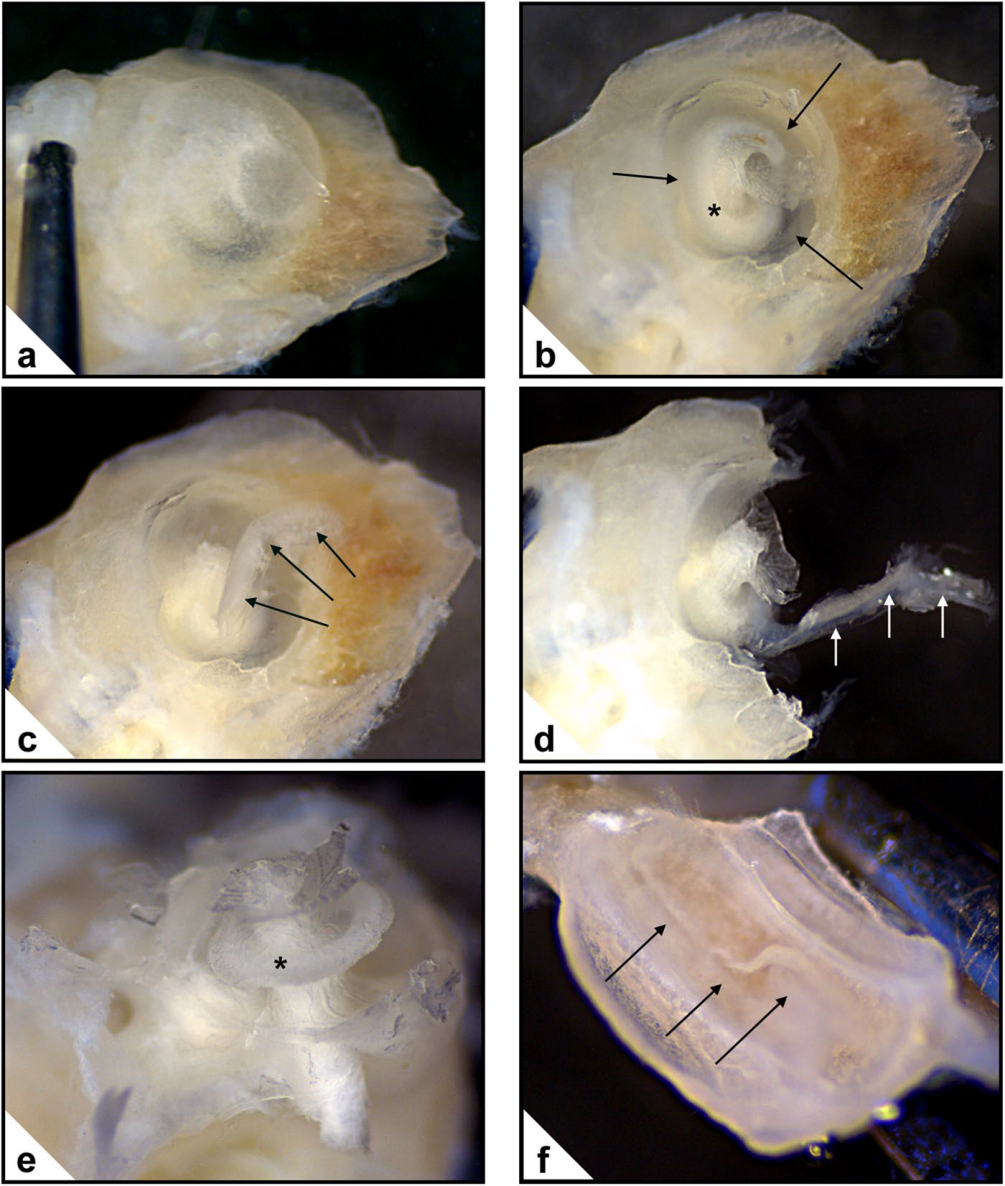
Dissection of the cochlea. (**a**) External aspect of the intact bony cochlea. (**b**) Bony wall of the cochlea partly removed. Cochlear duct: →; osseus spiral lamina: *. (**c**) Removal of the apical part of the cochlear duct (→). (**d**) Cochlea broken into two parts for gaining access to the basal part of the cochlear duct (→). (**e**) The main part with the modiolus after removal of the cochlear duct. Osseus spiral lamina: *. (**f**) The other part of the cochlea with the cochlear duct (→). Subsequently this part of the cochlear duct will also be dissected (not shown).

**Figure 8:**
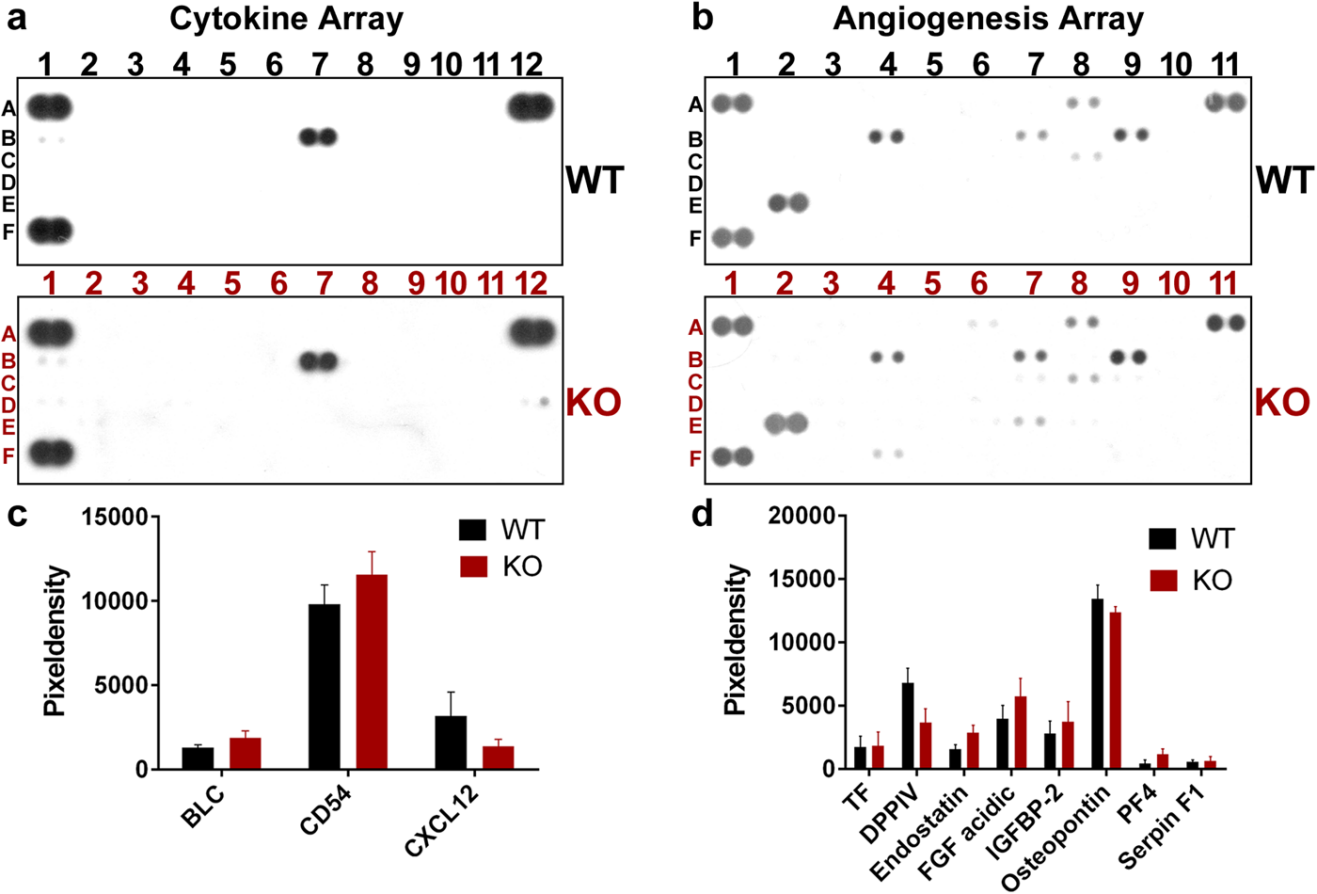
Proteome profile analysis of cochlear duct lysates with R&D Mouse Proteome Profiler Mouse Cytokine Array Panel A (a,c) and Mouse Angiogenesis Array Kit (**b**,**d**). (**a**,**b**) Representative images for Cytokine Array Panel A (B1: BLC, B7: CD54, D12: CXCL12) and Angiogenesis Array Kit (A8: TF, B4: DPPIV, B7: endostatin, B9: FGF acidic, C8: IGFBP-2, E2: osteopontin, E7: PF4, F4: serpin F1). See Supplementary Table S2 for identification of all measured proteins. (**c**,**d**) Pixel density of protein dots acquired from cochlear duct lysates. There were no significant differences in either examined factors between wild-type (WT) and PACAP-deficient (KO) mice. Mean ± SEM, Student’s t-test.

We used Proteome Profilers from the R&D Systems to elucidate the protein composition of the cochlear ducts of WT and PACAP KO mice (Figs 7, 8). The Mouse Cytokine Array Panel A and the Mouse Angiogenesis Array Kits are eligible to detect 40 and 53 different proteins, respectively. From the lysates of cochlear ducts endostatin, acidic FGF, osteopontin, BLC, CD54, PF4, TF, DPPIV, IGFBP-2, Serpin F1 and CXCL12 were in detectable quantity. The pixel density of the respective spots did not show significant differences between the WT and PACAP KO groups (Fig. 8, Supplementary Table S2).

## Discussion

Hearing impairment is a disease affecting approximately 500 million people worldwide. Hearing loss could be of various origins, including genetic disorders, intrauterine damage, contagious diseases, noise trauma, aging or toxic agents^44^. After our earlier studies in the inner ear, our present study further elucidates the role of endogenous PACAP in the auditory pathway and could lead to further investigations on the use of PACAP in different auditory system deficiencies.

The presence of PACAP has been demonstrated in the nuclei of the auditory pathway. It is present in the cell bodies and fibres of cochlear nuclei, with higher expression in the VCN^21,22^. It has been found in different nuclei of the SOC in rat and Djungarian hamsters, being described in the perikarya of medial nucleus of the trapezoid body, MSO, LSO and periolivary nuclei, including the nuclei of the olivocochlear fibres which could be the source of PACAP positive fibres in the cochlea^22,45^. PACAP was found in the medial olivocochlear tract ending on the outer hair cells but not in the lateral olivocochlear tract, which terminates on the dendrites of the spiral ganglion neurons under the inner hair cells. This confirms the theory that PACAP has a greater role in the efferent than in the afferent innervation of the cochlea^19^. PACAP was also found in the IC and medial geniculate body in rat and human brains^21,46^. PAC1 receptor mRNA expression was found in VCN, DCN, SOC, NLL, IC and medial geniculate body in the rat brain^47–49^. Human studies showed no PAC1 receptor expression in the IC under normal circumstances but its weak expression was present in sudden infant death syndrome brain samples^50^.

Based on these results, we examined the changes of the hearing function in PACAP KO animals. We found with ABR hearing threshold tests that PACAP KO mice have a significantly worse hearing with click stimuli and at 4.1 and 8.2 kHz frequencies compared to the WT ones at 1.5 and 4 months of age but there are no differences in the hearing thresholds at higher frequencies. At 8 months of age, the difference was significant at click and 4.1 kHz burst stimuli. With the evaluation of the hearing thresholds, it has to be taken into account that the CD1 mouse strain is subject to age related hearing loss. The hearing threshold pattern found in our experiments coincides with earlier described patterns characteristic for CD1 mice^51^. This could be the reason that there is no difference in the hearing threshold at higher frequencies and cause the disappearance of the significance at 8.2 kHz between the PACAP KO and WT mice at the age of 8 months. Although PACAP KO animals have no obvious deficit in hearing thresholds compared to WT animals in the higher frequency range tested (16.4; 32.8 and 65.6 kHz), amplitude and latency examinations revealed altered hearing functions at these frequencies. The amplitudes – in line with the activation and firing of the neurons in the hearing pathway – of the PACAP KO mice showed a strong decrease compared to WT littermates. However, the latencies – the time of the response after the stimulus - of the PACAP-deficient mice showed a bit faster neuronal activation and transmission. Decreased amplitude and latency values are probably the subtle indications of hearing impairment not revealed by hearing threshold measurements^52,53^. During the analysis of individual waves, the labelling of the peaks of PACAP-deficient mice was often cumbersome because of peak fusions. In KO mice, we saw fusion of peaks III and IV, however, these ABR waves in WT animals were rather separated.

To confirm the functional findings, we performed c-Fos immunolabeling to show the neuronal activation of the nuclei of the auditory pathway. Unlike Fos-B, c-Fos is a short-term activation marker of nerve cells commonly used in the auditory pathway resulting in alterations at gene expression level^54,55^. The activation was achieved by applying 4 to 20 kHz noise with 100 dB sound pressure for half an hour for the mice. Several different setups could be used for c-Fos activation measurements in the auditory pathway^56,57^, however, in our model we allowed 1 hour for the expression and nuclear translocation of c-Fos.

In the VCN and DCN, we found very few c-Fos immunopositive cells in the silent environment in both groups. However, there were activated neurons both in the WT and PACAP KO animals after noise exposure but the activation was significantly smaller in the KO mice. These results correspond to the functional ABR findings. Regarding the central parts of the auditory pathway, there was baseline cell activation in the SOC, NLL, IC and primary auditory cortex (AU1). The number of activated cells was significantly elevated after noise exposure in the SOC, NLL and IC in both genotype groups and elevation was also detected in the AU1 region but it was non-significant. However, we did not find any difference between the WT and PACAP KO groups. The significant differences between the silent and noise-exposed groups show that our measurements are valid and the lack of difference between the two genotypes does not result from a technical error. The reason for the baseline activation of neurons could arise from the fact that these nuclei are not simply relay stations of the auditory pathway but that they obtain afferentation from other brain areas and serve more complex functions. SOC receives innervation from IC, from thalamus, from AU1 and from serotonergic and noradrenergic centres; NLL from IC^58,59^. IC gains information from auditory and somatosensory cortices and from the somatosensory nuclei of the brainstem^60^. AU1 has a leading role in multimodal information processing^61^. These connections can explain the baseline neuronal activity in the silent animal groups and the disappearance of the differences between the WT and KO animal groups. It has long been hypothesized that changes in other signalling pathways could play a role in compensating for the lack of PACAP in PACAP-deficient animals^34,62^. We assume that there is a greater possibility for this compensation in the central nuclei of the auditory pathway as those obtain more complex connection patterns with several neuronal types releasing numerous neurotransmitters in these areas.

To elucidate the cause of the impaired hearing functions we aimed to further explore the role of endogenous PACAP in the inner ear, and therefore we performed proteome profile analysis from cochlear duct lysates from WT and PACAP-deficient mice. As mentioned in the introduction, PACAP and its specific PAC1 receptor have been demonstrated earlier in several parts of the inner ear^16^. After showing that PACAP had antiapoptotic effects on inner ear cells *in vitro*^23^, our research group also demonstrated that the levels of Ca^2+^-binding proteins (parvalbumin, calretinin, calbindin) are elevated in the hair cells of PACAP KO animals compared to WT ones^17^. We also described elevated Ca^2+^-binding protein levels in WT animals after ototoxic kanamycin treatment^43^. We suppose that the elevation of Ca^2+^-binding proteins in PACAP KO animals under normal circumstances could be a compensatory mechanism indicating pathological conditions in the inner ear. This pathology could be the result of the missing antiapoptotic and oxidative stress attenuating effects of PACAP in these animals. However, the baseline elevated Ca^2+^-binding protein levels in PACAP KO animals did not increase further after kanamycin treatment possibly indicating a limitation of this compensatory mechanism.

Inflammation^63,64^ and angiogenesis^65^ are known processes involved in different types of sensorineural hearing loss. In this study, we investigated their role in hearing loss that developed in the absence of endogenous PACAP. The use of isolated cochlear duct lysates excludes the contamination by proteins from the bony cochlea. We detected the ubiquitous cell surface enzyme DPPIV, antiapoptotic osteopontin, intercellular adhesion molecule CD54 and several other proteins taking part in angiogenic (acidic FGF, CXCL12), antiangiogenic (endostatin, Serpin F1), chemotactic (BLC, PF4, CXCL12) and coagulation (PF4, TF) procedures in both WT and PACAP-deficient mice. However, we did not find significant differences in the expression of the measured proteins between the two genotypes. We concluded that the differences in hearing between the WT and PACAP KO mice cannot be substantiated by alterations in the expression of the investigated proteins in the endolymphatic ducts. Results of the chosen proteome profiler kits do not support the involvement of inflammatory or angiogenic processes in the hearing impairment caused by the lack of PACAP.

It still remains an open question whether the hearing loss is caused by inner ear or auditory pathway lesions. It is known that isolated inner ear lesions also lead to complex changes in the auditory pathway. Besides the direct effect of the absence of neuronal activation in the nuclei of the auditory pathway, it could also lead to alterations in the tyrosine hydroxylase or Ca^2+^-binding protein expression^66,67^. The lesion of the inner ear alone could be the reason of the impaired hearing function and attenuated c-Fos expression in PACAP KO animals. Nissl staining also confirmed this, whereby in spite of the smaller number of activated c-Fos expressing neurons, the cell number of neurons in the VCN and DCN did not change significantly in PACAP KO animals compared to the WT ones.

We confirmed that CD1 mice have a progressive hearing loss by aging. This process involves the loss of inner and outer hair cells in the inner ear^51^. It is also known that aging processes are accelerated in PACAP KO mice affecting several organ systems^14,25,39–41^. An acceptable hypothesis could be that the changes we found in the auditory system of the PACAP KO mice are the result of the accelerated aging of the inner ear and auditory pathway. The neuroprotective and general cytoprotective effects of PACAP are not present in PACAP KO animals that could be the cause for an increased hair cell loss and consequent impaired hearing functions. However, hearing loss and the accompanying hair cell damage is present in the WT CD1 animals^51^, it could be only more expressively present in KO mice. This could be the reason why we did not detect significant differences in the proteome profile of WT and PACAP KO mice.

We conclude that endogenous PACAP is essential for normal hearing functions. The normal aging and the processes of hearing loss in the inner ear are accelerated in PACAP-deficient mice, but the exact mechanisms are yet to be elucidated. Still, the possibility arises that administration of PACAP or an agonist of its receptors may have a curative effect in age-related or noise-/ototoxic-drug-induced hearing loss, as we have already shown PACAP protection in another sensory system^13,68^. Therefore, we plan to elucidate the effects of ototoxic insults in PACAP KO animals and examine whether the impairments could be prevented by exogenous PACAP/agonist administration bringing us one step closer to reveal the curative effects of PACAP.

## Materials and Methods

### Animals

All the experiments were performed on male WT (CD1) and homozygous PACAP KO mice. Animals were fed and watered ad libitum, under light/dark cycles of 12/12 h. All procedures were performed in accordance with the ethical guidelines of the University of Pecs (approved by the Food Chain Safety and Animal Health Directorate, Government Office of Baranya County, Hungary, BA02/2000-24/2011) and Semmelweis University (approved by the Food Chain Safety and Animal Health Directorate, Government Office of Pest County, Hungary, XIV-I-001/1028-4/2012; PE/EA/1912-7/2017). The PACAP KO mice were generated by Hashimoto and co-workers^26^. Maintaining of the in-house-bred animals included backcrossing for 10 generations with CD1 mice. For details on genotyping, please see Supplementary Information.

### *In vivo* recordings of auditory brainstem responses (ABRs)

In mice and humans alike, sound-evoked potentials from the auditory brainstem appear as a series of consecutive peaks and valleys of ABR waves. These peaks come from the synchronous synaptic activity of nuclei along the afferent auditory pathway (Fig. 1/a). The first peak arises from the cochlea / spiral ganglion neurons ~1 ms after the stimulus onset (latency). Peaks II-V arise from the upper nuclei (i.e. cochlear nuclei, medial nucleus of the trapezoid body, SOC and IC) of the auditory pathway^69^. ABR measurements were performed as described before^70^. Briefly: mice were anaesthetized by intraperitoneal (i.p.) injections of ketamine (100 mg/kg, CP-Ketamin 10% injection, Produlab Pharma B.V., Netherland) and xylazine (10 mg/kg, CP-Xylazine 2% injection, Produlab Pharma B.V., Netherland). Ketamine-xylazine affects the results of ABR measurements compared to awake mice but it has less influence on the results than other commonly used anaesthetics (*e.g*. isoflurane)^71,72^. Body mass based calculation of anaesthetics and the same experimental protocol used for ABR measurements provided equal level of anaesthesia for all animals excluding its deteriorating effect on the KO vs. WT comparisons. During the ABR measurement, the body temperature was maintained and corneal drying was prevented. ABRs were recorded by an ABR System 3 workstation (Tucker-Davis Technologies, Alachua, FL). Click (0.4 ms duration) and tone burst (3 ms duration, 0.2 ms rise/decay; 4.1, 8.2, 16.4, 32.8 and 65.6 kHz) stimuli were generated by the SigGen software package and delivered in a closed acoustic system to the external auditory meatus through a plastic tube connected to an EC1 electrostatic speaker. Electroencephalograms were recorded with subdermal needle electrodes (Rochester Electro-medical Inc., USA) as the potential difference between an electrode on the vertex (active) and an electrode behind the right pinna (reference). The rear leg served as a ground. Measurements were always performed on the right ear. The evoked responses were amplified, and 800 sweeps were averaged in real time. The intensity was increased in 10 dB steps from 0 to 80 dB in click stimulation mode. To obtain auditory thresholds at different frequencies, the sound intensity of the tone burst stimuli was attenuated in 10 dB steps from 90 to 10 dB. The threshold was defined as the lowest intensity at which a visible ABR wave was seen (BioSig software) (Fig. 1/a). Numbers of measurements: 29 WT and 26 KO mice at the age of 1.5 month, 18 WT and 15 KO mice at the age of 4 months and 17 WT and 8 KO mice at the age of 8 months.

Besides measuring the hearing thresholds, we analysed the individual ABR waves evoked by tone burst stimuli in a Firebird based custom database in all of the 1.5-month-old mice (n = 29 WT and 26 KO). We identified the ABR peaks (peak I to peak V) and measured the amplitudes and latencies for each peak on each measured SPL. Peak latencies were determined relative to the onset of the acoustic stimuli and wave amplitudes were calculated as the difference between the two values represented by response maxima (peak) and subsequent minima (valley) (Figs 2/b, 3/b).

### Auditory pathway activation – c-Fos immunolabeling

We performed c-Fos immunolabeling for elucidating neuronal activation after white noise application in 1.5-month-old mice (n = 9 WT and 8 KO) in the following nuclei of the auditory pathway: VCN, DCN, SOC, NLL, IC and AU1^73^. We placed our mice into a closed ventilated “noise box”. Noise exposure (4 to 20 kHz white noise with 100 dB sound pressure, 30 min) was generated with the Audacity computer software (Dominic Mazzoni, GNU GPL license) and an amplifier (Mc.Taatoo, Nightline Pro 400) connected to the PC. The piezo high frequency speaker was located on the ceiling of the box. The overall noise level and frequency composition were measured using a calibrated microphone in a combination with SVAN971 (SVANTEK SP. Z O.O.) sound level meter. After noise exposure, we allowed 1 hour for c-Fos transcription, translation and for its subsequent translocation to the nucleus. In contrast to the noise exposed group (labelled as ‘noise’), the control group (labelled as ‘silent’) was held in a silent environment for 24 hours before termination including a 30 min period in the “noise box” without noise exposure.

Animals were overanesthetized with double-dose intraperitoneal ketamine-xylazine injection. Transcardial perfusion was performed by PBS solution (sodium phosphate-buffered saline, 20 ml, 0.1 M, pH 7.4), followed by paraformaldehyde fixative (150 ml 4% paraformaldehyde in 0.2 M Millonig sodium phosphate buffer, pH 7.4). After decapitation, the dissected brains were postfixed in the same fixative for 24 h, then 30 µm coronal sections were prepared using a Leica VT1000S vibratome (Leica Biosystems, Wetzlar, Germany). We performed free-floating immunohistochemistry with polyclonal rabbit anti-c-Fos antiserum (Santa Cruz Biotechnology Inc., sc-52, Santa Cruz, CA, USA, 1:500) followed by biotinylated goat anti-rabbit secondary antibody (Vectastain Elite ABC Kit, Vector Laboratories, Burlingame, CA, USA, 1:200)^27^. For visualization diaminobenzidine (DAB, D5637, Sigma-Aldrich, Hungary) was used. For detailed protocol please see Supplementary Information. For evaluation, a Nicon Microphot FXA microscope was used with a Spot RT colour digital camera (Nikon, Tokyo, Japan). Microphotographs were evaluated with ImageJ 1.50i program. For publication purposes, representative images were contrasted and in some cases the brightness was adjusted to correct the uneven illumination in peripheral areas. Touch-up tools were used to remove technical artefacts (i.e. dye crystals/particles, rests of embedding material) from the images (Photoshop 7.0.1, Adobe, San Jose, CA, USA). Digital editing did not influence the scientific message of the images.

### Cell count measurement - Nissl staining

For cell count measurements, the samples of untreated WT and KO animals (n = 3 WT and 3 KO) were fixed and sectioned as described for c-Fos immunolabeling. Then the sections were mounted on gelatine-coated glass slides and stained by Nissl (0.5 g Azur III; 0.5 g sodium tetraborate; 0.5 g toluidine blue; 30 g saccharose in 100 ml distilled water). Excess stain was removed by 96% and absolute alcohol. After xylene treatment (10 min), samples were coverslipped with DePex (Fluka, Heidelberg, Germany). Digitalization, evaluation and the correction of representative images were performed as described above. Cells with well-defined nuclei and nucleoli were counted to exclude glial cells from the measurement. Hearing impairment was similarly present in all three examined age groups (ABR). Therefore, the comparison of cell numbers was performed only in 4-month-old WT and KO mice.

### Cochlear duct proteome profile analysis

The bony cochlea was dissected from both sides of WT and PACAP KO mice (n = 20 WT and 20 KO) and placed in perilymph-like solution (composition in mM: NaCl 22.5; KCl 3.5; CaCl_2_ 1; MgCl_2_ 1; HEPES.Na 10; Na-gluconate 120; glucose 5.55; pH 7.4; 320 mOsm/l), which was continuously oxygenated^74^. Dissection of the cochlea occurred under operating microscope in the perilymph-like solution. The outer bony wall of the cochlea was removed and the apical part of the cochlear duct was separated from the modiolus. Then the cochlea was broken into two parts and the remaining part of cochlear duct was dissected (Fig. 7). The cochlear ducts were placed in Eppendorf tubes containing PBS buffer with protease inhibitor, homogenized with a handheld homogenizer, sonicated on ice for 2 min and centrifuged for 5 min with 14,000 rpm. Until further work, the samples were stored at −20 °C in the protease inhibitor containing solution. The protein concentrations of tissue samples were measured by absorption spectrophotometry. The samples were then analysed with R&D Systems Proteome Profiler Mouse Cytokine Array Panel A and Mouse Angiogenesis Array Kit (R&D Systems, Biomedica, Hungary) and handled according to the manufacturer’s description. The Proteome Profilers are able to detect 40–53 different proteins based on the specific binding between the antibodies (attached to a nitrocellulose membrane) and the proteins in the examined samples (Supplementary Table S2). The analyses were performed as described in the previous work of our research group^75^. Briefly: the samples were placed in buffer with 15 μl of primary antibody (1:100) and incubated for 1 h on room temperature. Meanwhile, the nitrocellulose membranes were blocked for 1 h. Thereafter, the reconstituted Detection Antibody Cocktail was placed on each membrane (1.5 ml pro membrane) and incubated for overnight on a moving platform at 2–8  °C. The membranes were washed with PBS for 3 × 10 min, treated with horseradish-peroxidase-conjugated streptavidin and washed again for 3 × 10 min in PBS. Then the membranes were treated with 2 ml of chemiluminescence detection reagent (Amersham Biosciences, Hungary). On the developed X-ray films the pixel volume of the spots correlates with the quantity of the corresponding proteins. The scanned images of the X-ray films were evaluated with ImageJ 1.50i software. The pixel volumes were normalized to the positive controls. All measurements arise from 2 independent kits providing results from 4 independent measurements (2 membranes per kit were used for the same group of animals). All individual samples contained the cochlear duct homogenates of 5–5 animals. Representative images were corrected to obtain optimal contrast (Photoshop 7.0.1, Adobe, San Jose, CA, USA). Hearing impairment was similarly present in all three examined age groups (ABR). Therefore, comparison of proteome profiles was performed only in 3–6-month-old WT and KO mice.

### Statistical analysis

Two-way ANOVA was performed and followed by Bonferroni post-hoc test for ABR hearing threshold, amplitude and latency as well as for c-Fos immunolabeling measurements. The homogeneity of variance as well as the normal distribution of datasets subjected to ANOVA was verified by Bartlett’s Chi-square and Shapiro-Wilk test, respectively. Two-tailed Student’s t-test was performed for Nissl staining and proteome profile analysis measurements. ‘N-1’ Chi-squared test was performed for the occurrence of peak IV measurements. Alpha level was 0.05 for all tests. Values are given as mean ± SEM. Statistica v8.0 (StatSoft Inc., USA), Prism 6.1 (GraphPad Software, USA) and Microsoft Excel (Microsoft Corporation, USA) were used for statistical analysis.

## Supplementary information

Supplementary Information

## Data Availability

The datasets and original digital images generated during and/or analysed during the current study are available from the corresponding authors on reasonable request.

## References

[1] Miyata A (1990). Isolation of a neuropeptide corresponding to the N-terminal 27 residues of the pituitary adenylate cyclase activating polypeptide with 38 residues (PACAP38). Biochem. Biophys. Res. Commun..

[2] Vaudry D (2009). Pituitary adenylate cyclase-activating polypeptide and its receptors: 20 years after the discovery. Pharmacol. Rev..

[3] Botia B (2007). Neurotrophic effects of PACAP in the cerebellar cortex. Peptides.

[4] *Pituitary Adenylate Cyclase Activating Polypeptide — PACAP*. (eds Reglodi, D. & Tamas, A.) **11**, (Springer, Cham, 2016).

[5] Ohtaki H, Nakamachi T, Dohi K, Shioda S (2008). Role of PACAP in ischemic neural death. J Mol Neurosci.

[6] Reglodi D, Vaczy A, Rubio-Beltran E, MaassenVanDenBrink A (2018). Protective effects of PACAP in ischemia. J. Headache Pain.

[7] Reglodi D, Kiss P, Lubics A, Tamas A (2011). Review on the protective effects of PACAP in models of neurodegenerative diseases *in vitro* and *in vivo*. Curr. Pharm. Des..

[8] White SL, May V, Braas KM (2000). Organization of the rat PACAP gene. Ann. N. Y. Acad. Sci..

[9] Waschek JA (2002). Multiple actions of pituitary adenylyl cyclase activating peptide in nervous system development and regeneration. Dev. Neurosci..

[10] Hansel DE, May V, Eipper BA, Ronnett GV (2001). Pituitary adenylyl cyclase-activating peptides and alpha-amidation in olfactory neurogenesis and neuronal survival *in vitro*. J. Neurosci..

[11] Wojcieszak J, Zawilska JB (2014). PACAP38 and PACAP6-38 exert cytotoxic activity against human retinoblastoma Y79 cells. J. Mol. Neurosci..

[12] Shioda S (2016). Pleiotropic and retinoprotective functions of PACAP. Anat. Sci. Int..

[13] Atlasz, T. *et al*. Protective effects of PACAP in the retina. In *Pituitary Adenylate Cyclase Activating Polypeptide –* PACAP (eds Reglodi, D. & Tamas, A.) 501–29 (Springer, Cham, 2016).

[14] Vaczy A (2018). Protective role of endogenous PACAP in inflammation-induced retinal degeneration. Curr. Pharm. Des..

[15] Lucero, M. T. Sniffing out a role for PACAP in the olfactory system. In *Pituitary Adenylate Cyclase Activating Polypeptide –* PACAP (eds Reglodi, D. & Tamas, A.) 483–99 (Springer, Cham, 2016).

[16] Fulop, B. D., Reglodi, D., Nemeth, A. & Tamas, A. Pituitary adenylate cyclase-activating polypeptide in the auditory system. In *Pituitary Adenylate Cyclase Activating Polypeptide –* PACAP (eds Reglodi, D. & Tamas, A.) 529–49 (Springer, Cham, 2016).

[17] Tamas A (2012). Comparative examination of inner ear in wild type and pituitary adenylate cyclase activating polypeptide (PACAP)-deficient mice. Neurotox. Res..

[18] Kawano H, Shimozono M, Tono T, Miyata A, Komune S (2001). Expression of pituitary adenylate cyclase-activating polypeptide mRNA in the cochlea of rats. Brain Res. Mol. Brain Res..

[19] Drescher MJ (2006). Pituitary adenylyl cyclase-activating polypeptide (PACAP) and its receptor (PAC1-R) are positioned to modulate afferent signaling in the cochlea. Neuroscience.

[20] Abu-Hamdan MD (2006). Pituitary adenylyl cyclase-activating polypeptide (PACAP) and its receptor (PAC1-R) in the cochlea: Evidence for specific transcript expression of PAC1-R splice variants in rat microdissected cochlear subfractions. Neuroscience.

[21] Hannibal J (2002). Pituitary adenylate cyclase-activating peptide in the rat central nervous system: An immunohistochemical and *in situ* hybridization study. J. Comp. Neurol..

[22] Kausz M, Murai Z, Arimura A, Koves K (1999). Distribution of pituitary adenylate cyclase activating polypeptide (PACAP) immunoreactive elements in the brain stem of rats studied by immunohistochemistry. Neurobiology (Bp)..

[23] Racz B (2010). PACAP ameliorates oxidative stress in the chicken inner ear: An *in vitro* study. Regul. Pept..

[24] Reglodi D (2012). PACAP is an endogenous protective factor-insights from PACAP-deficient mice. J. Mol. Neurosci..

[25] Maasz G (2014). Comparative protein composition of the brains of PACAP-deficient mice using mass spectrometry-based proteomic analysis. J. Mol. Neurosci..

[26] Hashimoto H (2001). Altered psychomotor behaviors in mice lacking pituitary adenylate cyclase-activating polypeptide (PACAP). Proc. Natl. Acad. Sci. USA.

[27] Gaszner B (2012). The behavioral phenotype of pituitary adenylate-cyclase activating polypeptide-deficient mice in anxiety and depression tests is accompanied by blunted c-Fos expression in the bed nucleus of the stria terminalis, central projecting Edinger-Westphal nucleus. Neuroscience.

[28] Matsuyama S, Matsumoto A, Hashimoto H, Shintani N, Baba A (2003). Impaired long-term potentiation *in vivo* in the dentate gyrus of pituitary adenylate cyclase-activating polypeptide (PACAP) or PACAP type 1 receptor-mutant mice. Neuroreport.

[29] Isaac ER, Sherwood NM (2008). Pituitary adenylate cyclase-activating polypeptide (PACAP) is important for embryo implantation in mice. Mol. Cell. Endocrinol..

[30] Lajko A (2018). Comparative analysis of decidual and peripheral immune cells and immune-checkpoint molecules during pregnancy in wild-type and PACAP-deficient mice. Am. J. Reprod. Immunol..

[31] Yamada K (2010). Increased stathmin1 expression in the dentate gyrus of mice causes abnormal axonal arborizations. PLoS One.

[32] Juhasz T, Helgadottir SL, Tamas A, Reglodi D, Zakany R (2015). PACAP and VIP signaling in chondrogenesis and osteogenesis. Peptides.

[33] Sandor B (2016). Structural and morphometric comparison of lower incisors in PACAP-deficient and wild-type mice. J. Mol. Neurosci..

[34] Fulop BD (2019). Altered Notch signaling in developing molar teeth of pituitary adenylate cyclase-activating polypeptide (PACAP)-deficient mice. J. Mol. Neurosci..

[35] Nakamachi T (2010). Endogenous pituitary adenylate cyclase activating polypeptide is involved in suppression of edema in the ischemic brain. Acta Neurochir. Suppl..

[36] Szakaly P (2011). Mice deficient in pituitary adenylate cyclase activating polypeptide (PACAP) show increased susceptibility to *in vivo* renal ischemia/reperfusion injury. Neuropeptides.

[37] May V, Vizzard MA (2010). Bladder dysfunction and altered somatic sensitivity in PACAP-/- mice. J. Urol..

[38] Markovics A (2012). Pituitary adenylate cyclase-activating polypeptide plays a key role in nitroglycerol-induced trigeminovascular activation in mice. Neurobiol. Dis..

[39] Reglodi D (2018). Accelerated pre-senile systemic amyloidosis in PACAP knockout mice - a protective role of PACAP in age-related degenerative processes. J. Pathol..

[40] Reglodi D (2018). PACAP deficiency as a model of aging. GeroScience.

[41] Kovacs-Valasek A (2017). Accelerated retinal aging in PACAP knock-out mice. Neuroscience.

[42] Ohtaki H (2010). Regulation of oxidative stress by pituitary adenylate cyclase-activating polypeptide (PACAP) mediated by PACAP receptor. J. Mol. Neurosci..

[43] Nemeth A (2014). Examination of calcium-binding protein expression in the inner ear of wild-type, heterozygous and homozygous pituitary adenylate cyclase-activating polypeptide (PACAP)-knockout mice in kanamycin-induced ototoxicity. Neurotox. Res..

[44] Sheffield, A. M. & Smith, R. J. H. The epidemiology of deafness. *Cold Spring Harb. Perspect. Med*. a033258 (2018). 10.1101/cshperspect.a033258 PMC6719589 30249598

[45] Reuss S (2009). Neurochemistry of olivocochlear neurons in the hamster. Anat. Rec..

[46] Palkovits M, Somogyvari-Vigh A, Arimura A (1995). Concentrations of pituitary adenylate cyclase activating polypeptide (PACAP) in human brain nuclei. Brain Res..

[47] Hashimoto H (1996). Distribution of the mRNA for a pituitary adenylate cyclase-activating polypeptide receptor in the rat brain: An *in situ* hybridization study. J. Comp. Neurol..

[48] Joo KM (2004). Distribution of vasoactive intestinal peptide and pituitary adenylate cyclase-activating polypeptide receptors (VPAC1, VPAC2, and PAC1 receptor) in the rat brain. J. Comp. Neurol..

[49] Shioda S (1997). Localization and gene expression of the receptor for pituitary adenylate cyclase-activating polypeptide in the rat brain. Neurosci. Res..

[50] Huang J, Waters KA, Machaalani R (2017). Pituitary adenylate cyclase activating polypeptide (PACAP) and its receptor 1 (PAC1) in the human infant brain and changes in the Sudden Infant Death Syndrome (SIDS). Neurobiol. Dis..

[51] Le Calvez S, Avan P, Gilain L, Romand R (1998). CD1 hearing-impaired mice. I: Distortion product otoacoustic emission levels, cochlear function and morphology. Hear. Res..

[52] Tziridis K, Buerbank S, Eulenburg V, Dlugaiczyk J, Schulze H (2017). Deficit in acoustic signal-in-noise detection in glycine receptor α3 subunit knockout mice. Eur. J. Neurosci..

[53] Muniak MA, Ayeni FE, Ryugo DK (2018). Hidden hearing loss and endbulbs of Held: Evidence for central pathology before detection of ABR threshold increases. Hear. Res..

[54] Brown MC, Liu TS (1995). Fos-like immunoreactivity in central auditory neurons of the mouse. J. Comp. Neurol..

[55] Riera-Sala C, Molina-Mira A, Marco-Algarra J, Martínez-Soriano F, Olucha FE (2001). Inner ear lesion alters acoustically induced c-Fos expression in the rat auditory rhomboencephalic brainstem. Hear. Res..

[56] Lu J (2014). Antioxidants reduce cellular and functional changes induced by intense noise in the inner ear and cochlear nucleus. J. Assoc. Res. Otolaryngol..

[57] Takagi H, Saito H, Nagase S, Suzuki M (2004). Distribution of fos-like immunoreactivity in the auditory pathway evoked by bipolar electrical brainstem stimulation. Acta Otolaryngol..

[58] Thompson AM, Schofield BR (2000). Afferent projections of the superior olivary complex. Microsc. Res. Tech..

[59] Thompson AM, Thompson GC (1993). Relationship of descending inferior colliculus projections to olivocochlear neurons. J. Comp. Neurol..

[60] Bajo VM, King AJ (2013). Cortical modulation of auditory processing in the midbrain. Front. Neural Circuits.

[61] Budinger E, Laszcz A, Lison H, Scheich H, Ohl FW (2008). Non-sensory cortical and subcortical connections of the primary auditory cortex in Mongolian gerbils: Bottom-up and top-down processing of neuronal information via field AI. Brain Res..

[62] Reglodi D (2018). Disturbed spermatogenic signaling in pituitary adenylate cyclase activating polypeptide-deficient mice. Reproduction.

[63] Kalinec GM, Lomberk G, Urrutia RA, Kalinec F (2017). Resolution of cochlear inflammation: Novel target for preventing or ameliorating drug-, noise- and age-related hearing loss. Front. Cell. Neurosci..

[64] Koles L, Szepesy J, Berekmeri E, Zelles T (2019). Purinergic signaling and cochlear injury-Targeting the immune system?. Int. J. Mol. Sci..

[65] London NR, Gurgel RK (2014). The role of vascular endothelial growth factor and vascular stability in diseases of the ear. Laryngoscope.

[66] Idrizbegovic E, Bogdanovic N, Canlon B (1998). Modulating calbindin and parvalbumin immunoreactivity in the cochlear nucleus by moderate noise exposure in mice. A quantitative study on the dorsal and posteroventral cochlear nucleus. Brain Res..

[67] Tong L, Altschuler RA, Genene Holt A (2005). Tyrosine hydroxylase in rat auditory midbrain: Distribution and changes following deafness. Hear. Res..

[68] Endo K (2011). Neuroprotective effect of PACAP against NMDA-induced retinal damage in the mouse. J. Mol. Neurosci..

[69] Melcher JR, Kiang NY (1996). Generators of the brainstem auditory evoked potential in cat. III: Identified cell populations. Hear. Res..

[70] Polony G (2014). Protective effect of rasagiline in aminoglycoside ototoxicity. Neuroscience.

[71] van Looij MAJ (2004). Impact of conventional anesthesia on auditory brainstem responses in mice. Hear. Res..

[72] Cederholm JME (2012). Differential actions of isoflurane and ketamine-based anaesthetics on cochlear function in the mouse. Hear. Res..

[73] Franklin, K. B. J. & Paxinos, G. *The mouse brain in stereotaxic coordinates*. (Academic Press, 2008).

[74] Berekmeri E (2019). Targeted single-cell electroporation loading of Ca^2+^ indicators in the mature hemicochlea preparation. Hear. Res..

[75] Horvath G (2010). Effects of PACAP on mitochondrial apoptotic pathways and cytokine expression in rats subjected to renal ischemia/reperfusion. J. Mol. Neurosci..

