# Supplementary material for: Hearing impairment and associated morphological changes in pituitary adenylate cyclase activating polypeptide (PACAP)-deficient mice
Source: Sci Rep. 2019 Oct 10;9:14598. doi: 10.1038/s41598-019-50775-z (PMC6787024; doi:10.1038/s41598-019-50775-z)
Supplement: Supplementary file 1 — Supplementary Information [file 41598_2019_50775_MOESM1_ESM.pdf]

Hearing impairment and associated morphological changes in pituitary adenylate cyclase activating polypeptide (PACAP)-deficient mice

Daniel Balazs Fulop, Viktoria Humli, Judit Szepesy, Virag Ott, Dora Reglodi, Balazs Gaszner, Adrienn Nemeth, Agnes Szirmai, Laszlo Tamas, Hitoshi Hashimoto, Tibor Zelles, Andrea Tamas

Amplitude

Frequency	Peak	Stimulus intensity	P value
16.4 kHz	I	90 dB SPL	p=0.0367
16.4 kHz	II	90 dB SPL	p=0.0039
16.4 kHz	IV	70 dB SPL	p=0.0406
16.4 kHz	IV	80 dB SPL	p=0.0042
16.4 kHz	IV	90 dB SPL	p<0.0001
16.4 kHz	V	80 dB SPL	p=0.0028
16.4 kHz	V	90 dB SPL	p=0.0002
32.8 kHz	I	90 dB SPL	p=0.0106
32.8 kHz	IV	70 dB SPL	p=0.0015
32.8 kHz	IV	80 dB SPL	p<0.0001
32.8 kHz	IV	90 dB SPL	p<0.0001
32.8 kHz	V	80 dB SPL	p=0.0022
32.8 kHz	V	90 dB SPL	p=0.0009
65.6 kHz	II	90 dB SPL	p=0.0006
65.6 kHz	IV	80 dB SPL	p<0.0001
65.6 kHz	IV	90 dB SPL	p<0.0001
65.6 kHz	V	90 dB SPL	p=0.0008

Latency

Frequency	Peak	Stimulus intensity	P value
16.4 kHz	I	50 dB SPL	p=0.0134
16.4 kHz	II	70 dB SPL	p=0.0021
16.4 kHz	V	70 dB SPL	p=0.0418
65.6 kHz	I	70 dB SPL	p=0.0211
65.6 kHz	II	70 dB SPL	p=0.0052
65.6 kHz	V	90 dB SPL	p=0.0274

Occurrence of peak IV

Frequency	Stimulus intensity	P value
16.4 kHz	50 dB SPL	p=0.0093
16.4 kHz	60 dB SPL	p=0.0389
16.4 kHz	80 dB SPL	p=0.0461
32.8 kHz	60 dB SPL	p=0.0126
32.8 kHz	70 dB SPL	p=0.0034
65.6 kHz	70 dB SPL	p=0.0246
65.6 kHz	80 dB SPL	p=0.0461

Supplementary Table S1. P values for amplitude and latency measurements and for the occurrence of separately identifiable peak IV at the auditory brainstem responses (ABR).

R&D Proteome Profiler Mouse Cytokine Array Panel A

	1	2	3	4	5	6	7	8	9	10	11	12
A	Pos. ctrl											Pos. ctrl
B	**BLC**	C5a	G-CSF	GM-CSF	CCL1	Eotaxin	**CD54**	IFNγ	IL-1α	IL-1β	IL-1ra	IL-2
C	IL-3	IL-4	IL-5	IL-6	IL-7	IL-10	IL-12	IL-13	IL-16	IL-17	IL-23	IL-27
D	CXCL10	CXCL11	CXCL1	M-CSF	CCL2	CCL12	CXCL9	CCL3	CCL4	CXCL2	CCL5	**CXCL12**
E	CCL17	TIMP-1	TNFα	TREM-1								
F	Pos. ctrl											Neg. ctrl

R&D Proteome Profiler Mouse Angiogenesis Array Kit

	1	2	3	4	5	6	7	8	9	10	11
A	Pos. ctrl		ADAMTS1	AR	ANG	Ang-1	Ang-3	**TF**	CXCL16		Pos. ctrl
B		Cyr6	DLL4	**DPPIV**	EGF	CD105	**Endostatin**	ET-1	**FGF acidic**	FGF basic	
C		KGF	CX3CL1	GM-CSF	HB-EGF	HGF	IGFBP-1	**IGFBP-2**	IGFBP-3	IL-1α	IL-1β
D		IL-10	IP-10	KC	Leptin	CCL2	CCL3	MMP-3	MMP-8	MMP-9	NOV
E		**Osteopontin**	PD-ECGF	PDGF-AA	PDGF-AB	PTX3	**PF4**	PIGF-2	PRL	Proliferin	
F	Pos. ctrl	SDF-1	Serpin E1	**Serpin F1**	TSP-2	TIMP-1	TIMP-4	VEGF	VEGF-B	Neg. ctrl	

Supplementary Table S2. Location of all examined proteins on the corresponding representative pictures in Fig. 8. Bold marking shows proteins in detectable amount. Abbreviations used according to the manufacturer’s operation manual.

Supplementary methods

Genotyping procedures

Phire Animal Tissue Direct PCR Kit (Thermo Fischer Scientific, Waltham, MA, USA) was used for genotyping from mouse tail samples. Primer sequences for detection of WT DNA signatures were 5′-ACC GAA AAC AAA TGG CTG TC-3′ (sense) and 5′-GGT CCA CAA AGTATATCT GTG CAT TCT-3′ (antisense); and for PACAP KO 5′-ATC TCC TGT CAT CTC ACC TTG CTC CT-3′ (sense) and 5′-GAA GAA CTC GTC AAG AGA GGC GAT AG-3′ (antisense). After PCR reaction and agarose gel electrophoresis the gels were stained with Sybr Green I (Sigma-Aldrich, Hungary). For evaluation, no template samples were used as negative control and heterozygous PACAP KO templates as positive control.

Free-floating immunohistochemistry

The sections were washed in 0.1 M sodium PBS solution for 3x10 min to remove the fixative solution. Thereafter, sections were incubated in 0.5% Triton X-100, then in PBS containing 2% normal goat serum (NGS, Jackson Immunoresearch Europe Ltd., Suffolk, UK) for 30-30 min. Overnight incubation followed with polyclonal anti-c-Fos antiserum (Santa Cruz Biotechnology Inc., sc-52, Santa Cruz, CA, USA, 1:500) in blocking buffer at room temperature. After 3x10 min PBS washes, sections were incubated with biotinylated goat anti-rabbit IgG (Vectastain Elite ABC Kit, Vector Laboratories, Burlingame, CA, USA, 1:200) in PBS containing 2% NGS at room temperature for 2 h. After PBS wash, sections were put in avidin-biotin complex solution (Vectastain Elite ABC Kit, Vector Laboratories, Burlingame, CA, USA) for 1 hour at room temperature then again rinsed with PBS for 3x10 min. For visualization 0.02% 3,3’ diaminobenzidine (DAB, D5637, Sigma-Aldrich, Hungary) was used in Tris buffer with H2O2 (0.00003%) for 10 min. Under visual control, the reaction was stopped with PBS. After 3x10 min PBS washes, sections were mounted on gelatine-coated glass slides and after xylene treatment (2x10 min) coverslipped with DePex (Fluka, Heidelberg, Germany). The specificity of the polyclonal antiserum against c-Fos was tested previously in mice by our research group using synthetic c-Fos blocking peptide (Santa Cruz Biotechnology Inc., sc-52 P, Santa Cruz, CA, USA) [1]. Preincubation of the antibody with the blocking peptide eliminated any positive reactions, as did the substitution of the anti-c-Fos serum by nonimmune rabbit serum.

1. Gaszner, B. *et al.* The behavioral phenotype of pituitary adenylate-cyclase activating polypeptide-deficient mice in anxiety and depression tests is accompanied by blunted c-Fos expression in the bed nucleus of the stria terminalis, central projecting Edinger-Westphal nucleus. *Neuroscience* **202**, 283–299 (2012).
